# Effects of COVID-19 Social Distancing Measures in Individuals with Chronic Pain Living in Spain in the Late Stages of the Lockdown

**DOI:** 10.3390/ijerph182211732

**Published:** 2021-11-09

**Authors:** Jordi Miró, Elisabet Sánchez-Rodríguez, Alexandra Ferreira-Valente, José Pais-Ribeiro, Antonella Ciaramella

**Affiliations:** 1 Research Center for Behavior Assessment (CRAMC), Unit for the Study and Treatment of Pain—ALGOS, Department of Psychology, Universitat Rovira i Virgili, 43007 Catalonia, Spain; elisabet.sanchez@urv.cat; 2 Institut d’Investigació Sanitària Pere Virgili, Universitat Rovira i Virgili, 43003 Catalonia, Spain; 3 William James Center for Research, ISPA—University Institute, 1100-304 Lisbon, Portugal; mafvalente@gmail.com (A.F.-V.); jlpr@fpce.up.pt (J.P.-R.); 4 Department of Rehabilitation Medicine, University of Washington, Seattle, WA 98195, USA; 5 Faculty of Psychology and Education Sciences, University of Porto, 4099-002 Porto, Portugal; 6 Lab. of Psychosomatic, GIFT Institute of Integrative Medicine, 56126 Pisa, Italy; ciarantogift@gmail.com

**Keywords:** COVID-19, social distancing measures, pain, fatigue

## Abstract

Social distancing measures during the lockdown have had a negative impact on chronic pain patients’ function. Research, however, has only focused on the early stages of the first lockdowns. The aim of this study was to improve the understanding of the effects of COVID-19 social distancing measures on individuals with chronic pain living in Spain during the late stages of the lockdown. A group of 361 adults with pain participated in this study. They responded to an online survey and provided information on sociodemographic issues, pain, fatigue, perceived health, and quality of life. The data showed that most participants suffered moderate to severe pain and interferences with pain treatment and an increase in pain intensity during the lockdown. Most participants also informed us that fatigue had worsened during the lockdown (62%). Importantly, females with lower monthly family income and lower education have been found to be associated with greater levels of pain and fatigue. Despite this, participants perceived themselves as having good health and good quality of life. The findings from this study can be used to inform policy and specific responses for future COVID-19 waves and future pandemics where social distancing measures must be implemented.

## 1. Introduction

The COVID-19 pandemic has impacted the lives of citizens worldwide, particularly those with pre-existing health conditions, including chronic pain [1]. 

Chronic pain is a public health problem that places an enormous burden on individuals and society [2]. The biopsychosocial model of chronic pain hypothesizes that pain and its impact are influenced by biological, psychological and social factors [3]. Although the influence of social factors has been studied less than the influence of biological and psychological factors [4], research has shown that social support is associated with the physical and psychological function of individuals with chronic pain. For example, in a study of young people with disabilities and chronic pain, Miró and colleagues [5] found that social support was negatively associated with physical function and fatigue. Similarly, Solé and colleagues found that the lack of social support and social isolation were related to depression in a sample of young adults with chronic pain [6]. Social isolation has consistently been found to be associated with a greater impact of pain on patients’ function and adjustment [7,8]. Moreover social support has been reported to be a protective factor for disability in this population [9,10]. Other variables in the social domain, such as economic status and education, have also been found to be associated with chronic pain [11,12].

Although social distancing measures during national lockdowns were necessary for COVID-19 to be controlled, research has shown that they are associated with an increased risk of problems for individuals with chronic pain. For example, in a survey on a sample of 1018 adults with migraine conducted in the early stages of the lockdown [13], the authors found a 60% increase in the frequency of pain episodes, and a transition from acute to chronic pain in 10% of the cases. Similarly, in a cross-sectional survey study with a sample of 150 patients with chronic pain, conducted in the early stages of the lockdown, Hruschak et al [14] found that social distancing was associated with greater pain severity and pain interference. Changes in daily routines related to the lockdown also contributed to greater emotional distress and fatigue in this population [15,16], which, in turn, were found to be associated with chronic pain-related interference [17]. 

During the early stages of the lockdown, patients did not always receive their treatment because healthcare professionals were primarily attending to COVID-19 related issues [18]. Furthermore, patients might have been less interested or willing to go to the hospital because they were afraid of being infected. Thus, it has also been suggested that the difficulties patients faced in following and receiving proper treatment for chronic pain during the lockdown are responsible, at least in part, for the worsening of their condition. For example, [19] found that restricted access to healthcare and increased dependence on others were associated with negative wellbeing outcomes in terms of sleep, anxiety and depression in a sample of adults with chronic pain. Unsupervised changes in treatment procedures to adapt to the new circumstances may have also contributed. For example, Nieto and colleagues found a significant increase in medication intake without any guidance from specialists in a sample of adults with chronic pain during the early stage of the lockdown [20], and unsupervised use of medication for pain has been shown to be related to problems in psychological and social function among chronic pain patients [21]. Finally, during the lockdown, many individuals could not continue working, which resulted in socio-economic problems and emotional dysfunction (see e.g., [22]). In turn, this has been associated with a worsening of chronic pain, in general (see e.g., [23]). Interestingly, Toprak and colleagues found that individuals who stayed at home during lockdown reported higher levels of low back pain than those who continued going to work [24].

Taken as a whole, the findings of these studies consistently indicate that the social distancing measures during the lockdown had a negative impact on chronic pain patients’ physical, psychological, and social function. To the best of our knowledge, all the studies have focused on the early stages of the first (national) lockdowns. Consequently, the suggestions made on how to improve the care of patients with chronic pain during a lockdown were based on what was known about the early stages of isolation to which everyone was exposed (e.g., [25]. However, they may not be applicable to the latter stages of lockdowns or subsequent lockdowns. In fact, it has been suggested that the impact of social distancing measures increases as lockdowns become longer [26]. In order to develop specific programs for preventing and managing the negative consequences of social distancing measures on the physical, psychological and social function of individuals with chronic pain, the impact of the longer stages of lockdown on the life of individuals also needs to be monitored. Furthermore, it is important to study what happens in subsequent stages of the pandemic as there have been various waves and it is still possible that there might be more. So social isolation may still be necessary. Importantly, it has been suggested that subsequent waves of recurrence may require social distancing to varying degrees in the years to come, possibly into 2022 [27]. 

Therefore, the main objective of this study is to improve the understanding of the effects of COVID-19 social distancing measures on individuals with chronic pain living in Spain during the late stages of the lockdown. In particular, we focused on how the measures affected their pain experience (specifically, the intensity of the pain episodes) and the treatment received/used for pain (specifically, the relief obtained), the fatigue they experienced, and any changes in pain and fatigue in comparison to the pre-lockdown situation. We also wanted to know how they perceived their health and quality of life, and we explored whether these variables (i.e., pain, fatigue, perceived health and quality of life) were associated with demographic (i.e., gender, age) and social characteristics (i.e., education, marital status, and monthly household income).

## 2. Methods 

### 2.1. Participants

Potential participants for this study came from a sample of 544 adults who had participated in an online survey that we conducted to study the impact of COVID-19 lockdown measures on the life of adults living in Spain. The inclusion criteria for the study were that the participants had to (1) be living in Spain during the lockdown; (2) be suffering from chronic pain; (3) have access to the Internet; (4) be 18 years old or older; (5) be able to read and write Spanish; and (6) provide their informed consent. In this study, following the definition of the International Association for the Study of Pain [28], chronic pain has been conceptualized as pain that has been present for at least three months. 

### 2.2. Procedure

To recruit the sample of participants, we used the social media of our research group (e.g., Facebook, Twitter) to publicize the study and distribute a link to our online survey. First of all, when participants entered the survey, they found a detailed explanation of the study and the informed consent page. In order to participate, interested individuals had to express their consent by clicking “YES” in response to a question about consent. The online survey was available for participation from 3 June–30 July 2020.

The study was approved by the Ethical Committee for Medical Research of the Pere Virgili Health Research Institute (ref. 117/2020).

### 2.3. Measures

Demographic and social characteristics of the sample. Participants were requested to inform us about their gender, age, education, marital status, province of residence, and monthly household income.

Pain. Participants were asked to report whether they had experienced pain during the lockdown and, if so, in which part of the body. Then, they were asked to report the duration of the pain problem using a 7-point verbal rating scale with alternatives ranging from “<than three months” to “≥ten years”, and the intensity of the pain. Participants were asked to report the mean intensity of their pain during the previous 24 hours using a numerical rating scale: that is, they had to give a number between 0 (“No pain”) and 10 (“Pain as bad as could be”). Pain intensity scores with numerical rating scales, like this one, have been shown to be valid when used with adults [29] including electronic versions [30,31,32]. Next, they were asked to report if the pain intensity had changed during the lockdown compared to the situation before the lockdown. They also had to report the extent to which social distancing had interfered with their ability to control the pain and the relief they had achieved with the treatment they had received/used for the pain.

Fatigue. In order to measure fatigue, we used the Silhouettes Fatigue Scale (SFS; [33]. The SFS is a visual scale that depicts six human figures or silhouettes showing an increasing level of fatigue. The scale is scored from 0 to 10, and has proved to provide valid information about fatigue with different samples [33,34,35]. Participants were asked to indicate the mean level of fatigue they had experienced during the lockdown. In addition, they were asked to report the current level of fatigue compared to the beginning of the lockdown, on a 5-point rating scale (from “I am much less fatigued” to “I am much more fatigued”). 

Perceived health and quality of life. Participants were asked the following questions, extracted from the 2010–2012 World Values Survey (Health Perception) [36]: “In general, how would you rate your health in the previous month?” and “In general, how would you rate your quality of life in the previous month?” to report on their health and quality of life, respectively. They had to respond on a 5-point verbal rating scale ranging from “Bad” to “Excellent”.

### 2.4. Data Analyses

We computed descriptive statistics (means and standard deviations for continuous variables and number and percentages for dichotomous variables) to describe the sample of participants and the study variables. In addition, to study whether differences in the outcome variables (i.e., pain, fatigue, perceived health and quality of life) were related to demographic (i.e., gender and age) and social (i.e., education, marital status, and monthly household income) characteristics of the sample, we used t-tests (for gender comparisons) and ANOVAS (for age [18–24 years old, 25–40, 41–65 and >65 years old], academic level [no studies or basic studies, secondary studies, and university studies], marital status [single, married or living with a partner, divorced and widow/widower] and monthly household income [≤EUR950, EUR951-EUR1900, EUR1901-EUR3800, EUR3801-EUR7600, EUR7601-EUR15200, ≥EUR15201]). We also computed Pearson correlations for continuous variables, Spearman correlations for ordinal variables, and Chi square tests for nominal variables. The analyses were performed using the Statistical Package for Social Sciences for Windows version 27.0 (SPSS Inc., Chicago, IL, USA).

## 3. Results

### 3.1. Description of the Study Sample and the Study Variables

A group of 361 adults with pain responded to the online survey, of whom 243 had chronic pain. The sample consisted mostly of a group of females (86%), with university studies (61%), who were married or living with their partner (52%), and with an average age of 40.95 years (SD = 15). Most participants were in the middle- and upper-class economic groups (67%). See Table 1, Table 2 and Table 3 for additional details of the sample and the study variables.

### 3.2. Effects of COVID-19 Social Distancing Measures on the Variables in the Study

In relation to pain, participants reported an average pain intensity of 5.08 (SD = 1.97), thus showing a tendency towards the upper end of the scale. A pain intensity of 5 on a 0–10 scale is considered to be moderate pain and deserving of treatment [37]. Importantly, they also reported interferences with pain treatment, and a significant number also reported an increase in pain intensity during the lockdown (N = 109; 45%). Similarly, although fatigue was reported to be in the lower range (see Table 2), most participants informed that fatigue had worsened during the lockdown (N = 149; 62%). Despite this, participants perceived themselves as having good health ($\bar{X}$ = 3.00; SD = 1.07) and a good quality of life ($\bar{X}$ = 2.94; SD = 0.98). 

### 3.3. Associations between the Study Variables and Demographic Characteristics of the Participants (i.e., Age and Gender)

In terms of gender, the *t*-test showed that there were only significant differences in pain intensity scores (*t* = 2.25, *p* = 0.029), with females reporting higher pain intensity. However, there were no statistically significant differences associated with age for any of the study variables (i.e., pain intensity, fatigue, perceived health, and quality of life).

### 3.4. Associations between the Study Variables and Social Characteristics of the Participants (i.e., Academic Level, Monthly Household Income, and Marital Status)

In terms of academic level, significant differences emerged in both perceived health (F = 4.18; *p* = 0.016) and quality of life (F = 4.60; *p* = 0.011). Participants with a university education reported a statistically significant better perceived health than individuals with no education or basic education (*p* = 0.045) and a better quality of life than individuals with secondary education (*p* = 0.020).

As far as monthly household income is concerned, statistically significant differences were found in fatigue (F = 2.80, *p* = 0.019) and perceived quality of life (F = 2.88, *p* = 0.016). Specifically, participants with a monthly household income between EUR 951 and 1900 reported significantly higher levels of fatigue than those with a monthly income between EUR 2801 and 7600 (*p* = 0.019). Furthermore, participants with a monthly household income between EUR 951 and 1900 and between EUR 1901 and 3800 reported significantly worse perceived quality of life than those with a monthly household income between EUR 7601 and 15,200 (*p*s < 0.05).

No differences were found in any of the study variables associated with marital status.

## 4. Discussion

The primary aim of this study is to improve our understanding of the effects of COVID-19 social distancing measures on individuals with chronic pain living in Spain during the late stages of the lockdown. In particular, we focused on how the measures affected their pain and the treatment received or used, the fatigue they experienced, and the changes in pain and fatigue in comparison to the pre-lockdown situation. We also wanted to know how they perceived their health and quality of life during that period, and to explore whether these variables (i.e., pain, fatigue, perceived health and quality of life) were associated with demographic (i.e., gender, age) and social characteristics (i.e., education, marital status, and monthly household income).

Two key findings emerged. First, pain intensity and fatigue were moderate and participants reported significant interferences in getting pain relief due to social distancing and the various public health measures implemented to prevent the pandemic from spreading in Spain. Importantly, a significant number of participants reported that pain intensity and fatigue had worsened during the lockdown. However, the perceived health and quality of life were reported to be good. Second, the data showed no age-related differences in any of the outcome variables (i.e., pain intensity, fatigue, perceived health and quality of life). However, female participants reported higher pain intensity scores, although gender was not found to affect the other outcome variables. Interestingly, participants with the highest education reported better perceived health and quality of life. Relatedly, individuals with the lowest monthly family income reported the highest fatigue and worst perceived quality of life. 

These findings are consistent with studies showing that during the lockdown individuals with chronic pain showed a decreased ability to self-manage pain and a restricted access to healthcare [19]. They are also in line with previous studies showing that social distancing measures during the lockdown are associated with a worsening in fatigue, pain intensity and difficulties in getting access to pain management among patients with chronic pain [14,25,38,39]. Furthermore, research has reported that the impact of the COVID-19 crisis on health has been greater in people with chronic pain than in the general population [40]. This study shows that this impact lasts, and that there is a need to develop specific strategies for this population. 

Taken as a whole, the findings thus far suggest that particular attention should be given to patients with chronic pain during emergency healthcare crises such as this one. Particularly for females, having a lower monthly family income and lower education have been found to be associated with greater pain and fatigue. 

This world health crisis has provided an opportunity to identify strategic needs and pinpointed basic inequities, including access to medical services and appropriate management of pain care, which should be promptly addressed [14]. For example, research has shown that specific services are required during lockdown to maintain independence and self-management so that wellbeing in this population can be preserved [19]. Studies need to identify the best practical solutions to the needs and difficulties that individuals with chronic pain face under these circumstances. In relation to this particular issue, the so-called new technologies (e.g., mobile apps) have been suggested as an alternative [41] although not all pain-related mobile apps have been developed on the basis of scientific tenets [42], and most have not been tested before dissemination [43]. Therefore, not all can be used safely [44]. However, studies show that mobile apps can effectively help to measure pain, (e.g., [30]), and self-manage different pain conditions, such as back pain [45], fibromyalgia [46] or cancer-related pain [47]. Mobile apps should be developed following available guides (e.g., [48], and undergo usability tests and efficacy studies. Future studies should also identify the barriers to the implementation of this technology and also the facilitators. Importantly, they need to identify how to provide patients with effective interdisciplinary treatment that ensures their privacy. 

Although this study offers valuable information regarding the experience of patients with chronic pain during social distancing, it has a number of limitations that should be considered when interpreting the results. First, although the number of participants was appropriate for the analyses that were conducted, and even greater than in other studies (e.g., [16], the sample size was relatively small. Additional research, ideally with larger sample sizes, would be needed to establish the validity of the findings. Second, the study design allowed concurrent associations among the study variables to be evaluated, but not causal associations. Moreover, we were not able to study if pain conditions were related to different effects. Future studies should address this gap, as it is imperative to develop a more comprehensive understanding about the impact of social distancing amongst different types of patients with pain in other geographic locations impacted by COVID-19. In particular, they need to identify those for whom it is most problematic. This information could lead to a better understanding of the impact of social isolation on pain and guide the development of specific approaches to support this vulnerable population in the case of potential subsequent waves of COVID-19 and new social distancing measures.

## 5. Conclusions

Despite the study’s limitations, the findings provide important additional information on the importance of the long-term effects of social confinement during lockdown on patients with chronic pain. Although social isolation may have provided opportunities for some, such as increased time with the family, for others, it has been a significant threat to their quality of life and wellbeing [18] 

The findings from this study can be used to inform policy and specific responses for future COVID-19 waves, and new future pandemics. This is particularly important as new waves are already swamping some world regions, and an increase in chronic pain after the COVID-19 pandemic is expected [49,50].

## Figures and Tables

**Table 1:** Descriptive data for the study sample (*n* = 243).

	Mean (SD)	*n*	%
**Age**	40.95 (15.00)		
18–24 years old		52	21
25–40 years old		65	27
41–65 years old		117	48
Over 65 years old		9	4
**Gender**			
Female		423	86
Male		121	14
**Education**			
No education or some basic education		7	3
Secondary education		88	36
University education		148	61
**Marital status**			
Single		92	38
Married or living with a partner		127	52
Divorced		23	9.5
Widow/widower		1	0.5
**Monthly family income**			
<EUR 950		6	4
EUR 951–1900		48	29
EUR 1901–3800		74	44
EUR 3801–7600		30	18
EUR 7601–15,200		2	1
>EUR 15,201		7	4

**Table 2:** Descriptive data for the study variables.

Domain.	Mean (SD)	Range	*n*	%
Pain intensity	5.08 (1.97)	0–10	243	
Interferences with treatment for pain	7.11 (3.70)	0–10		
Changes in pain intensity compared to pre-lockdown				
It has worsened a lot			26	11
It has worsened a bit			83	34
It has stayed the same			113	46
It has improved a bit			17	7
It has improved a lot			4	2
Fatigue	3.98 (2.63)	0–10		
Changes in fatigue compared to pre-lockdown				
Much more fatigued			55	23
A little more fatigued			94	39
It has stayed the same			59	24
A little less fatigued			29	12
Much less fatigued			6	2
Perceived health	3.00 (1.07)	1–5		
Quality of life	2.94 (0.98)	1–5		

**Table 3:** Associations between study variables.

	Sociodemographic Variables				
Study Variables	Age	Gender	Academic Level	Household Income	Marital Status
Pain intensity	0.07	0.15 *	−0.10	−0.16 *	33.83
Fatigue	−0.07	0.05	−0.10	−0.25 ***	19.58
Perceived health	0.018 **	0.07	−0.16 *	−0.18 *	9.94
Quality of life	−0.01	0.09	−0.20 **	−0.15	8.90

Note: We used Pearson correlations for age, Spearman correlations for gender, academic level, and household income, and chi-square tests for marital status; * *p* < 0.05; ** *p* < 0.01; *** *p* < 0.001.
